# Pathology in the appendicular bones of southern tamandua, *Tamandua tetradactyla* (Xenarthra, Pilosa): injuries to the locomotor system and first case report of osteomyelitis in anteaters

**DOI:** 10.1186/s12917-019-1869-x

**Published:** 2019-04-25

**Authors:** Leonardo Cotts, Roberta V. Amaral, Maíra Laeta, Carlos A. Cunha-Filho, Ricardo Moratelli

**Affiliations:** 1 Programa de Pós-graduação em Biodiversidade e Biologia Evolutiva da Universidade Federal do Rio de Janeiro (UFRJ), Instituto de Biologia, Centro de Ciências da Saúde (CCS), Interbloco B/C, Campus Ilha do Fundão, Av. Carlos Chagas Filho, 373, Cidade Universitária, Rio de Janeiro, RJ Brazil; 2 Fiocruz Mata Atlântica, Fundação Oswaldo Cruz (FIOCRUZ), Estrada Rodrigues Caldas, 3400 - Curicica, Rio de Janeiro, RJ Brazil; 3 Departamento de Geologia e Paleontologia, Museu Nacional, Universidade Federal do Rio de Janeiro (UFRJ), Quinta da Boa Vista, S, Rio de Janeiro, RJ /N Brazil; 4 Departamento de Vertebrados, Setor de Mastozoologia, Museu Nacional, Universidade Federal do Rio de Janeiro (UFRJ), Quinta da Boa Vista, S/N, Rio de Janeiro, RJ Brazil; 5 Grupo de Estudos de Mamíferos Marinhos da Região dos Lagos (GEMM-Lagos), Rio de Janeiro, RJ Brazil; 6 Programa de Pós-graduação em Ciências Ambientais e Conservação do Campus Macaé da Universidade Federal do Rio de Janeiro (UFRJ), Institute of Biodiversity and Sustainability at the Federal University of Rio de Janeiro (NUPEM/UFRJ), Av. São José Barreto, 764 - São José do Barreto, Macaé, RJ Brazil

**Keywords:** Anatomical pathology, Animal anatomy, Bone diseases, Myrmecophagidae, Vermilingua

## Abstract

**Background:**

The southern tamandua, *Tamandua tetradactyla* (Linnaeus, 1758), is the most common species of anteater. Even though much is known about its ecology, behavior, and parasites, there is very limited information about bone diseases in *Tamandua* and other anteaters. Here, we examined postcranial skeletons of 64 *T. tetradactyla* museum specimens covering most of the material available in Brazilian collections.

**Results:**

The following bone diseases were identified for the first time in *Tamandua* and other extant and fossil vermilinguans: osteophytes, osteitis, osteoarthritis, periostitis, exostoses, enthesopathies, and a severe chronic pyogenic osteomyelitis associated with fistulae, cloacae (pus), bone loss, and neoformation processes. Musculoskeletal reconstruction revealed that an old specimen was restricted to terrestrial locomotion due to osteopathological processes that impaired its climbing.

**Conclusions:**

New osteopathological informations are presented for *T. tetradactyla*, favoring a better understanding of the expression of some bone diseases in wild animals. In addition, the diagnosis of these bone diseases in living anteaters provides useful information for studies on animal health and welfare, as well as contributing to the more effective recognition of paleodiseases in fossil xenarthrans.

## Background

Mammals in the superorder Xenarthra are sister to Epitheria, comprising the most basal placental mammals [1], and are arranged into the orders Cingulata and Pilosa [2]. Cingulata includes New World armored mammals, whereas Pilosa includes sloths (suborder Folivora) and anteaters (suborder Vermilingua; [2]). Anteaters have peculiar anatomical features that distinguish them from all other mammals such as an elongated rostrum with a posteriorly displaced palate, incomplete zygomatic arch, a worm-like tongue attached in the xiphoid process of the sternum, absence of teeth and forelimbs with strong muscles adapted for the increase of force during the movement (e.g. The *Teres major* with its origin enlargement on the scapula and more distally inserted on the humerus) [3, 4]. Vermilingua are endemic to the Neotropics and widely distributed in South and Central America, including Trinidad [2]. The group comprises 10 extant species distributed in two families: Cyclopedidae, including the silky anteaters, with seven species — *Cyclopes catellus*, *C. didactylus*, *C. dorsalis*, *C. ida*, *C. rufus*, *C. thomasi*, and *C. xinguensis* [5]; and Myrmecophagidae, which comprises the giant anteater, *Myrmecophaga tridactyla*, and the northern and southern anteater, *Tamandua mexicana* and *T. tetradactyla*, respectively [2]. *Tamandua mexicana* occurs from Central America southward to northern South America, whereas *T. tetradactyla*, widely distributed in South America, is the most common anteater throughout its distribution range [2, 6].

Most investigations on anteaters have focused on their ecology and evolution (e.g. [7–10]), whereas pathological and osteopathological aspects are still poorly known and described mostly from soft tissue [11–13] and bone fractures [14]. Conversely, bone diseases are very common in humans and domestic animals such as dogs, cats, and horses, with osteomyelitis being one of the most recurrent injuries in the appendicular skeleton of these groups [15–19]. Osteomyelitis is an infection that commonly affects the long bones, causing inflammation and destruction of the bone and bone marrow [20, 21]. Different pathogens including bacteria, fungi, and viruses can infect the bone tissue and cause osteomyelitis, but the most frequent etiologic agent is the gram-positive bacteria *Staphylococcus aureus* [22, 23].

In this study, we surveyed for bone diseases in 64 appendicular skeletons of *Tamandua tetradactyla* housed in Brazilian collections (Collections details in **Methods – Material examined**). The analyzed specimens include males and females of different ages and from different localities, covering most of the postcranial material available for *Tamandua* in Brazil. The diseases found comprise biomechanical and inflammatory injuries, including the first case of chronic pyogenic osteomyelitis for anteaters. In addition, we also report evidence of forced terrestrial locomotion in *T. tetradactyla* as the result of an osteopathological process associated with the old age of the specimen examined.

## Results

### Description and diagnosis of diseases in the appendicular skeleton of *Tamandua tetradactyla*

Of the 64 *T. tetradactyla* skeletons examined, we found evidence of osteopathological lesions in four specimens (MN 79028, MN 79287, MZUSP 32329, and NPM 069). Diagnosis data are described in detail below.

**Fig. 1:**
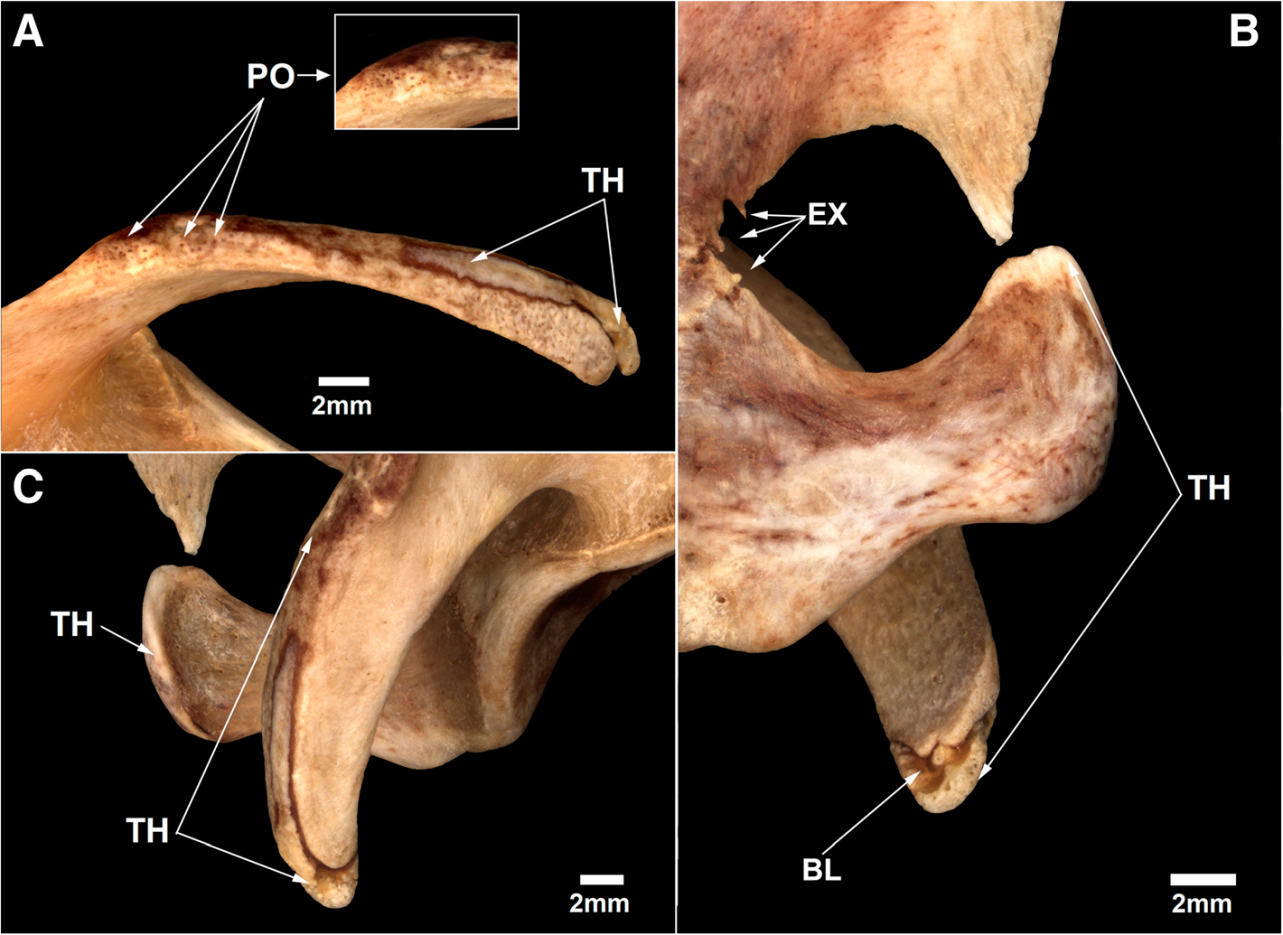
Bone thickening and bone remodeling in the left scapula of *Tamandua tetradactyla* (MN 79028). **a** Acromion process in dorsal view; **b** Scapular notch, Coracoid and acromion process in medial view; **c** acromion process in lateral view. BL, Bone loss; EX, Exostosis; PO, Porous region; TH, Thick region

The left scapula in specimen MN 79028 had some osteopathological conditions. The proximodorsal acromial surface has inconspicuous small pores (Fig. 1a), whereas the median and distal portions of the laterodorsal border are thick and have a slightly smooth surface (Fig. 1b). The medial surface of the distal portion of the acromion process of the left scapula has some concavities and an irregular bone surface, probably resulting from a lesion of inflammatory origin. The distal ends of the coracoids of the scapulae are also thick, especially in the dorsal portion (Fig. 1c). The more marked bone thickening observed in the distal regions of the coracoid and acromion process has a lighter coloration than the other portions of the scapula. The thickest regions in the scapula of MN 79028 are clearly denser compared to the same region from other *T. tetradactyla* specimens.

**Fig. 2:**
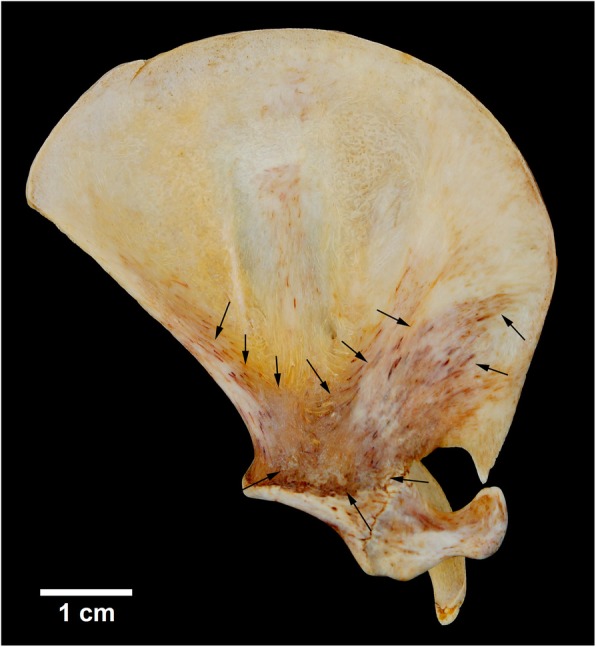
Marked darkening in ventromedial surface of the left scapula of *Tamandua tetradactyla* (MN 79028). Black arrows, darkened regions

Some very small pointed bone structures are found in the anterior region of the scapular neck of the left scapula of MN 79028, being projected anteriorly and into the scapular notch (Fig. 1b). These structures are osteophyte-like exostoses originated from abnormal bone growth (sensu [20, 22]). The left scapula of MN 79028, but not the right one, also has a marked darkening on the ventromedial surface and on the medial surface of the coracoid (Fig. 1b; Fig. 2). These darkened marks in the left scapula are superficial (extracortical) with no evidence of bone destruction or inflammation on radiographs. Thus, this condition probably resulted from a postmortem interaction of the scapula with the environment (e.g., necrolysis).

**Fig. 3:**
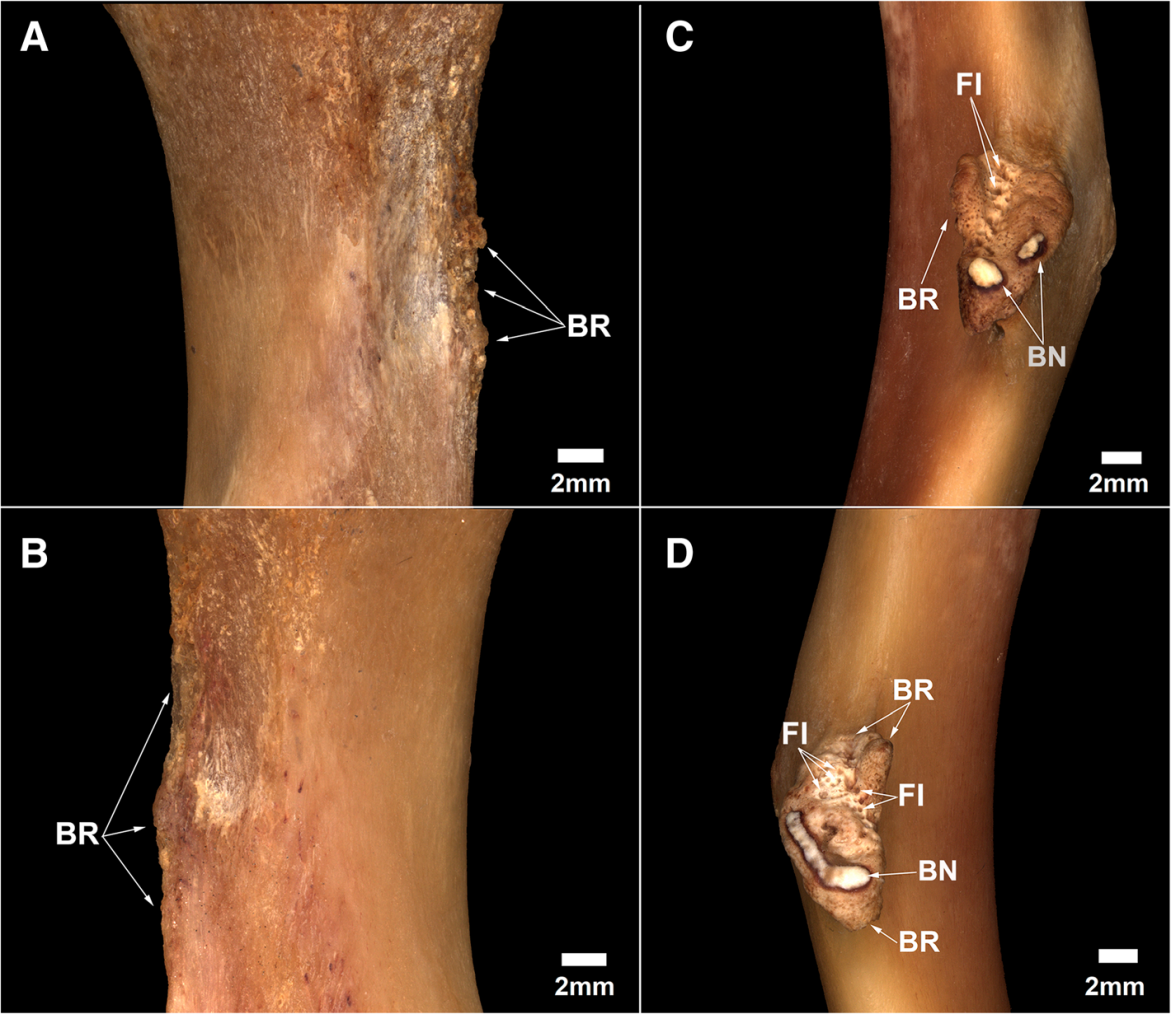
Signals of bone remodeling and inflammation in the humeri of *Tamandua tetradactyla* (MN 79028). **a**, **b** Right and left humeri in anterior view, respectively; **c**, **d** Deltoid tuberosity of the right and left humerus, respectively. BN, Bone neoformation; BR, Bone remodeling; FI, Fistulae

**Fig. 4:**
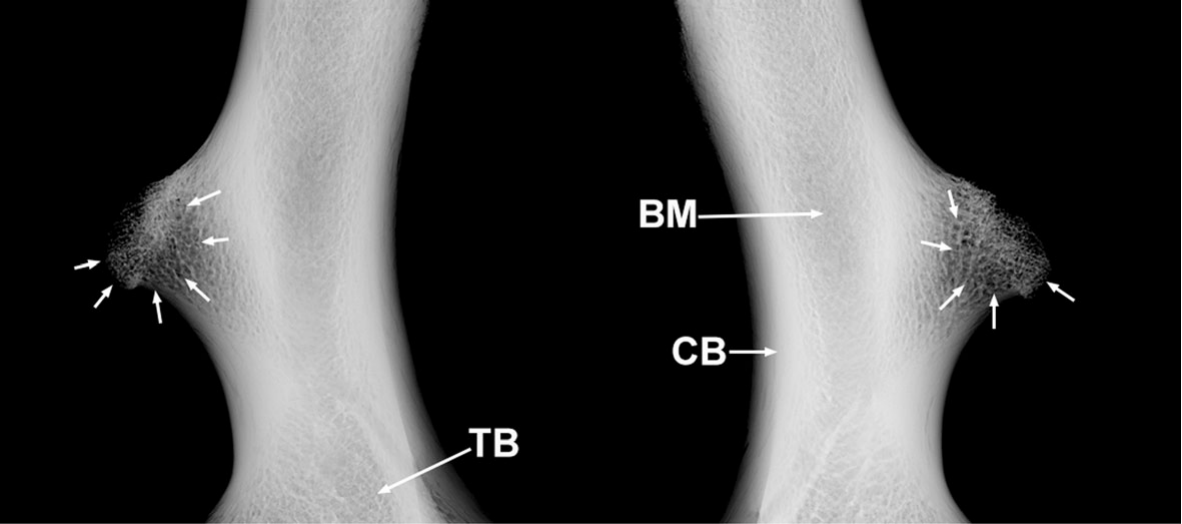
Radiographs of the injured humeri of *Tamandua tetradactyla* (MN 79028). BM, Bone marrow; CB, Cortical bone; TB, Trabecular bone. White arrows, radiolucent regions indicating bone resorption

The humeri of MN 79028 have marked pathological lesions. Some bone remodeling is observed in the medial margin of the proximal third of the humeri, with small bone neoformations associated (Fig. 3a and b). These lesions are more developed in the proximal third of the right humerus, with the medial margin showing an irregular and denticulate conformation. The lateral surface of deltoid tuberosities of the humeri has small pores and some bone remodeling, mainly in midproximal portions, whereas bone neoformations are found across almost the entire surface of this structure (Fig. 3c and d). Radiological analysis was used to reconstruct the progression of the lesion. Some well-marked radiolucent spaces were seen in the deltoid tuberosities of the humeri, deep inside the cortical bone (Fig. 4). The deltoid tuberosity of the left humerus has a slightly larger number of radiolucent spaces. The bone tissue inside the deltoid tuberosities has irregular shape, with small discontinuous channels, identified as fistulae, and areas of bone destruction (Fig. 4). Macrostructural and radiological analyses indicated that the regions mentioned above were affected by inflammation in the entheses (enthesopathy), probably due to repeated muscular effort throughout life. In the deltoid tuberosities, the enthesopathic lesions probably progressed to a more severe pathological condition, affecting the subperiosteal bone and causing osteitis [20, 22].

**Fig. 5:**
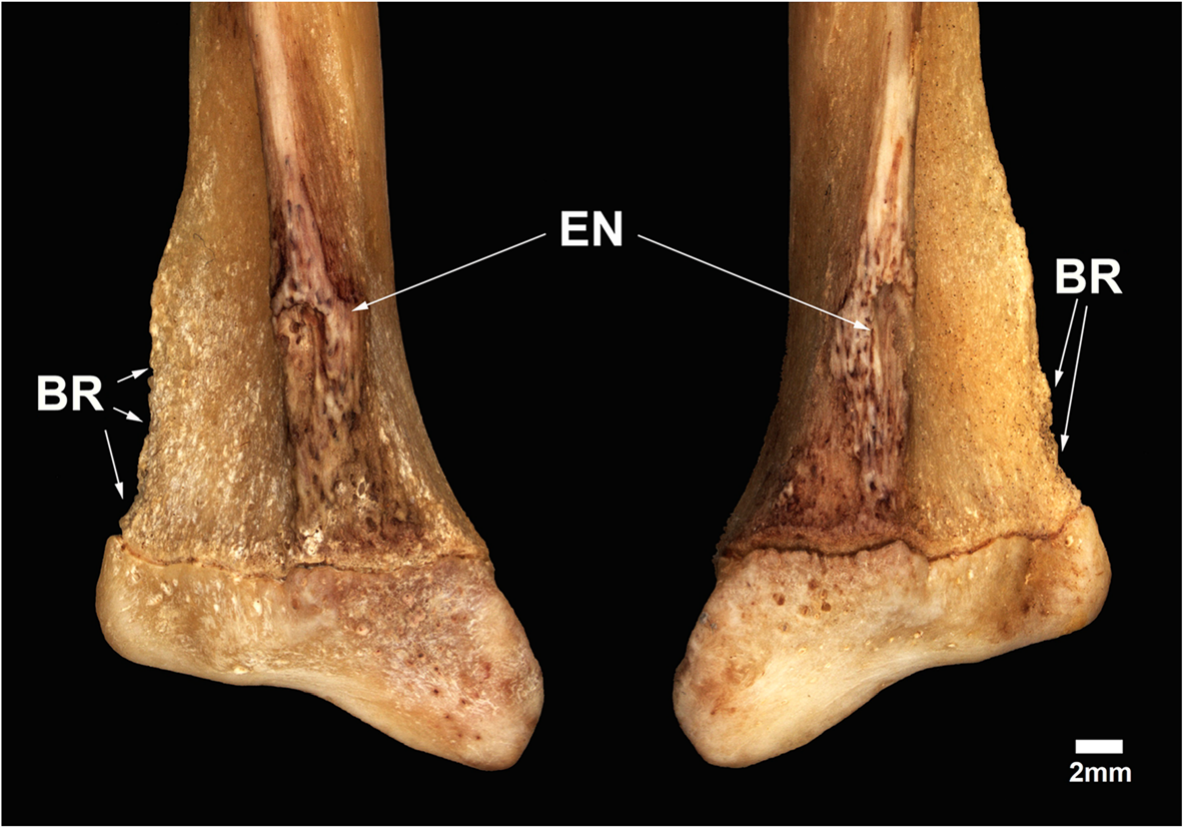
Distal third of the radii of *Tamandua tetradactyla* (MN 79028). **a**, **b** Right and left distal third of the radii in lateral view, respectively. BR, Bone remodeling; EN, Enthesopathy

**Fig. 6:**
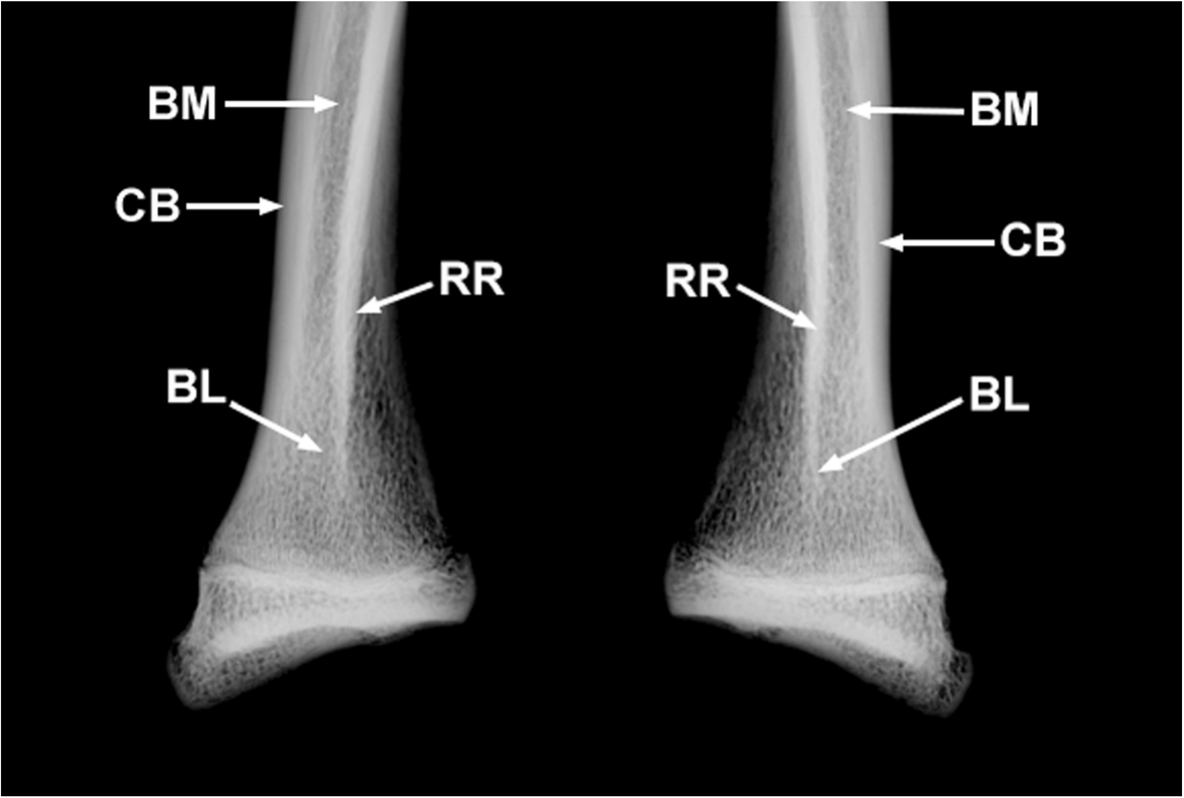
Radiographs of the injured distal third of the radii of *Tamandua tetradactyla* (MN 79028). BL, Bone loss; BM, Bone marrow; CB, Cortical bone; RR, Radial ridge

The radial ridges of the radii of MN 79028 has significant bone remodeling in distal portions, with a shallow and irregular concavity in the mid-distal region, and very small concavities scattered throughout the distal region distinct in appearance from the same region in healthy *T. tetradactyla* specimens (Fig. 5). In addition, the mid-distal region of the radial ridge of the radii of MN 79028 has a darker coloration, with small black dots scattered along its surface, distinct from that seen on the surface of the other regions of the radii from the same specimen (Fig. 5). Macroscopical and radiological analysis revealed the presence of bone lesions in the distal portion of the radial ridges, with destruction of the periosteal surface (Fig. 6). The lesions in the radial crests of MN 79028 were conclusively identified as enthesopathies, but in a more advanced stage than in the humerus from the same specimen (sensu [22]).

**Fig. 7:**
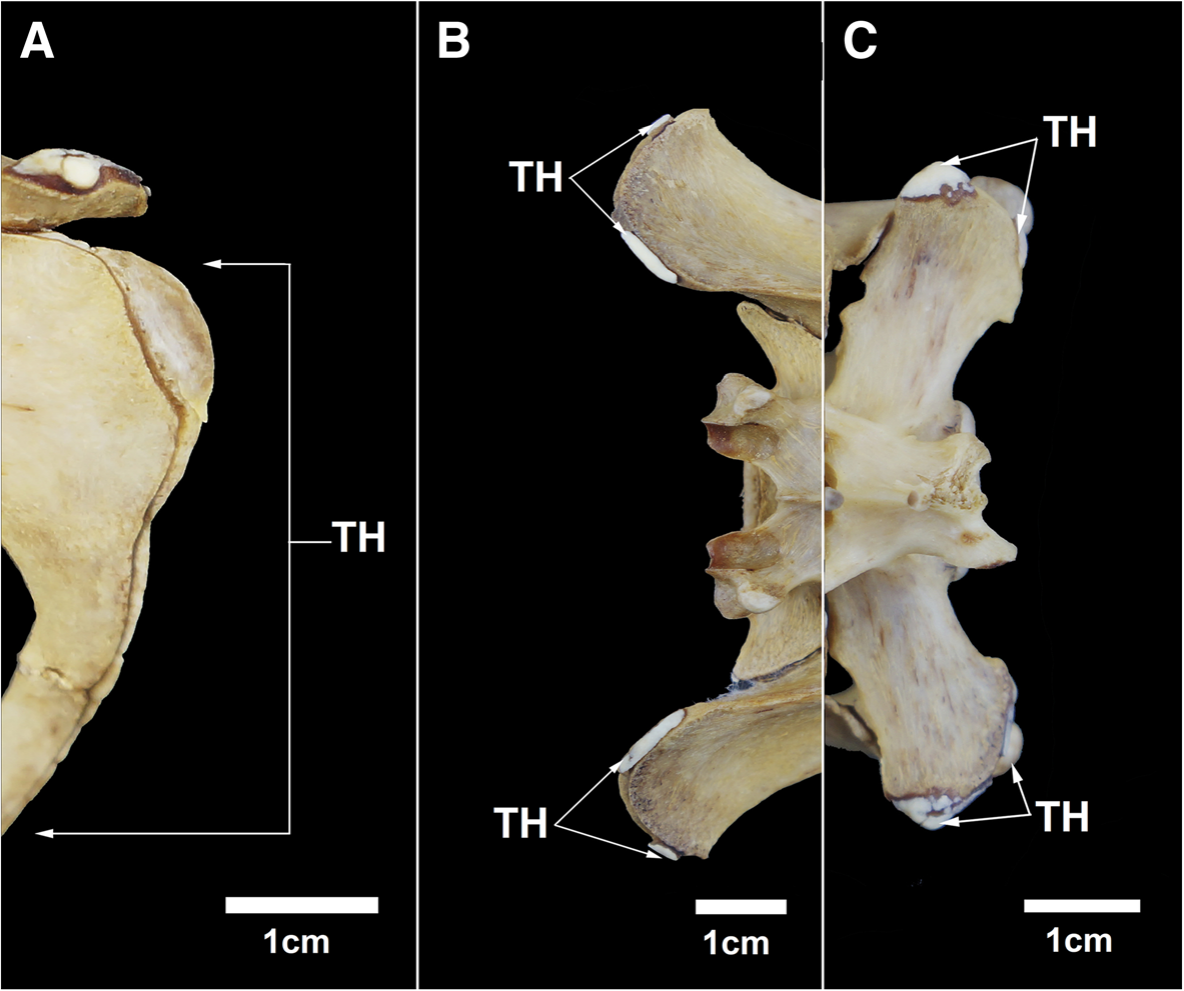
Bone thickening in the synsacrum of *Tamandua tetradactyla* (MN 79028). **a** Posterior margin of the synsacrum (ischium); **b** Anterodorsal margin of the synsacrum (ilium); **c** First caudal vertebra in dorsal view. TH, Thick region

The anterodorsal border (ilium) and posterior border (ischium) of the synsacrum of MN 79028 have some thick areas similar to those observed in the scapula from the same specimen (Fig. 7). Specimens MN 79287 and MZUSP 32329 also exhibit bone thickening in the same synsacral regions. In addition, the epiphyses of the long bones in MN 79287 and MZUSP 32329 shows heavy wear, with the surface totally porous in some regions and partially smooth in others.

**Fig. 8:**
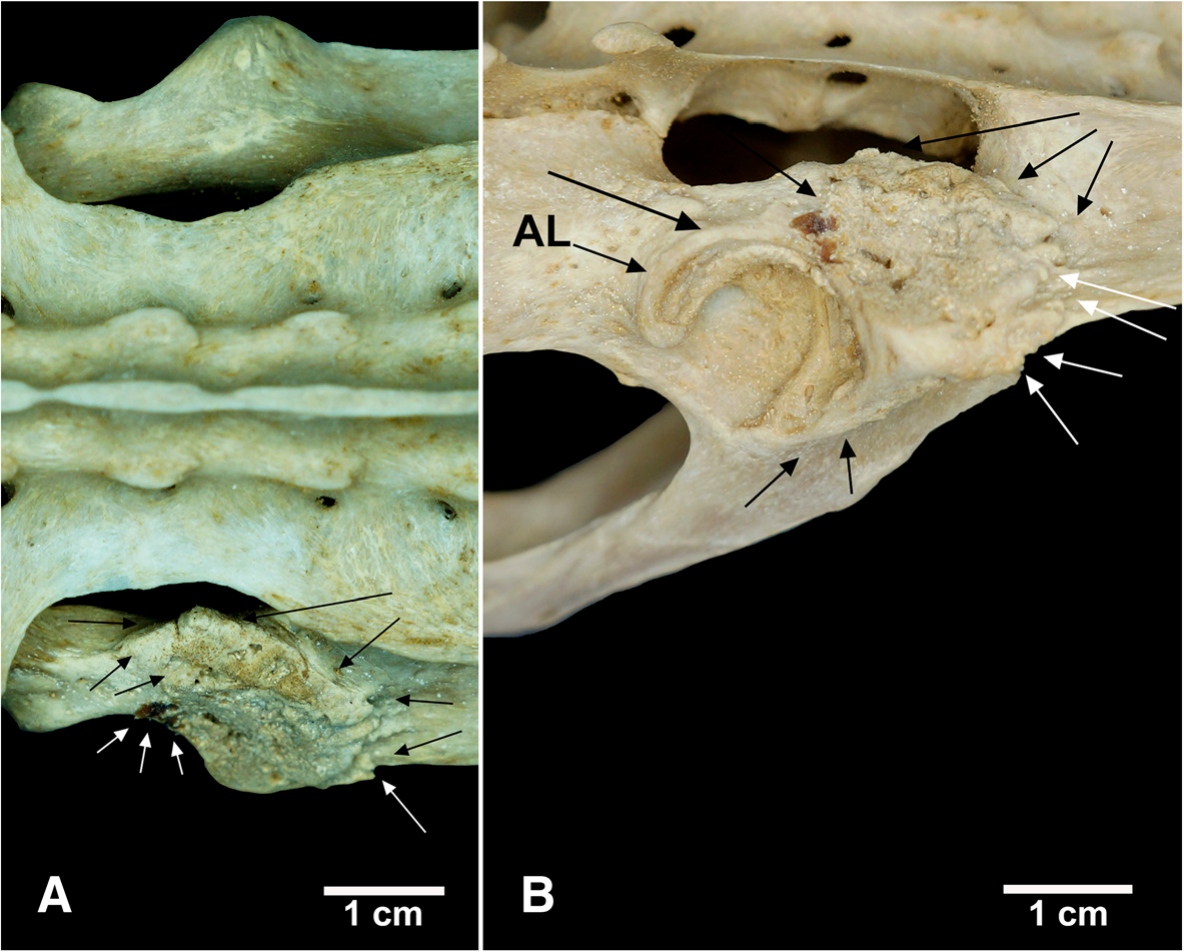
Bone remodeling in the right acetabulum of *Tamandua tetradactyla* (NPM 069). **a** Dorsal view; **b** lateral view. White arrows, osteophytes; Black arrows, intense bone remodeling, with processes of bone resorption and neoformation. AL, Acetabular labrum

Specimen NPM 069 has visible bone remodeling in the right acetabulum of the synsacrum (Fig. 8). The anterior portion of the acetabular labrum has irregular bone neoformations interspersed with marked bone resorption. The laterodorsal, lateroventral, and lateroanterior portions of the right acetabular labrum have increased bone thickening compared to the left acetabulum from the same specimen (Fig. 8). The anterodorsal portion of the right acetabulum is strongly remodeled, with well-developed bone neoformations and small grooves on its surface. The anterodorsal surface of the right acetabulum is asymmetrically dense and irregular in its margins. Some small osteophytes were seen in the anterior margin of this abnormal structure. Additional bone remodeling presenting as slight protuberances posteroventrally was seen in the peripheral region of the right acetabulum.

**Fig. 9:**
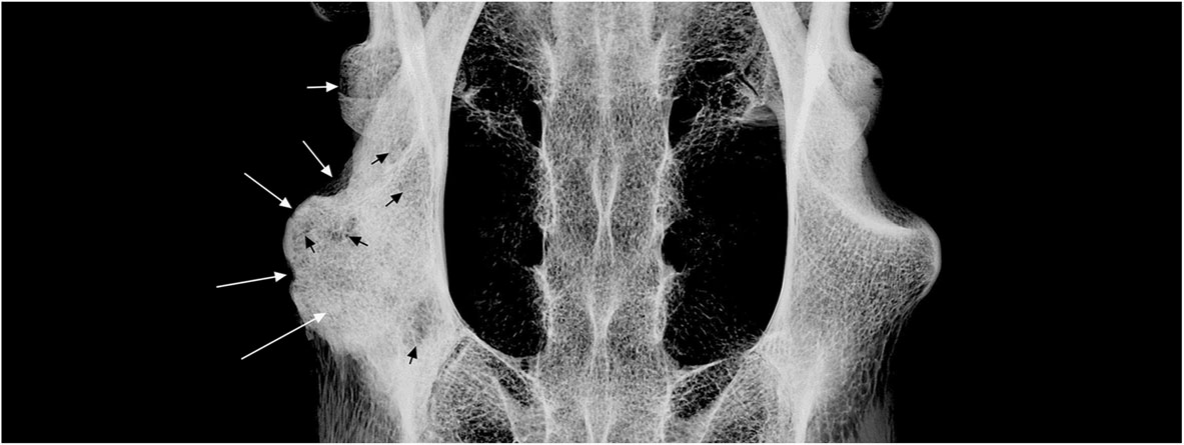
Radiograph of the right acetabulum of *Tamandua tetradactyla* (NPM 069**).** White arrows, bone remodeling; Black arrows, radiolucent regions indicating bone rarefaction

Radiological analysis revealed an irregular conformation of the trabecular bone of the right acetabulum in NPM 069 associated with heavy sclerosis of the subchondral bone (Fig. 9). Some radiolucent spaces were also observed laterally and medially in the diseased region of the acetabulum. The right acetabulum of NPM 069 shows evidence of an advanced inflammatory reaction.

**Fig. 10:**
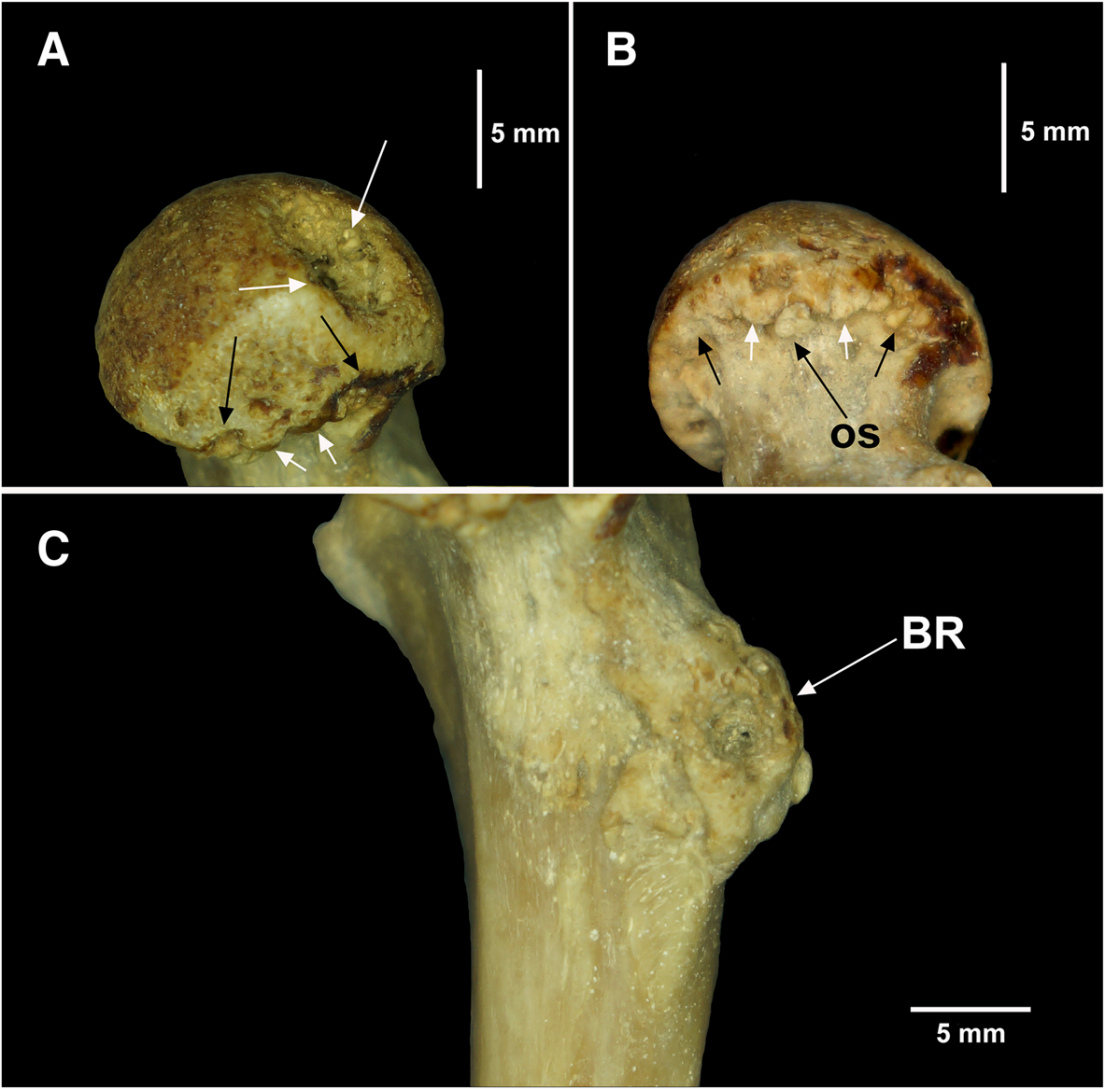
Proximal third of the right femur of *Tamandua tetradactyla* (NPM 069). **a** Dorsomedial view of the femoral head; **b** Ventromedial view of the femoral head; **c** Lesser trochanter of the femur in medial view. White arrows, Bone resorption; Black arrows, Bone neoformation. BR, Bone remodeling; OS, Osteophytes

**Fig. 11:**
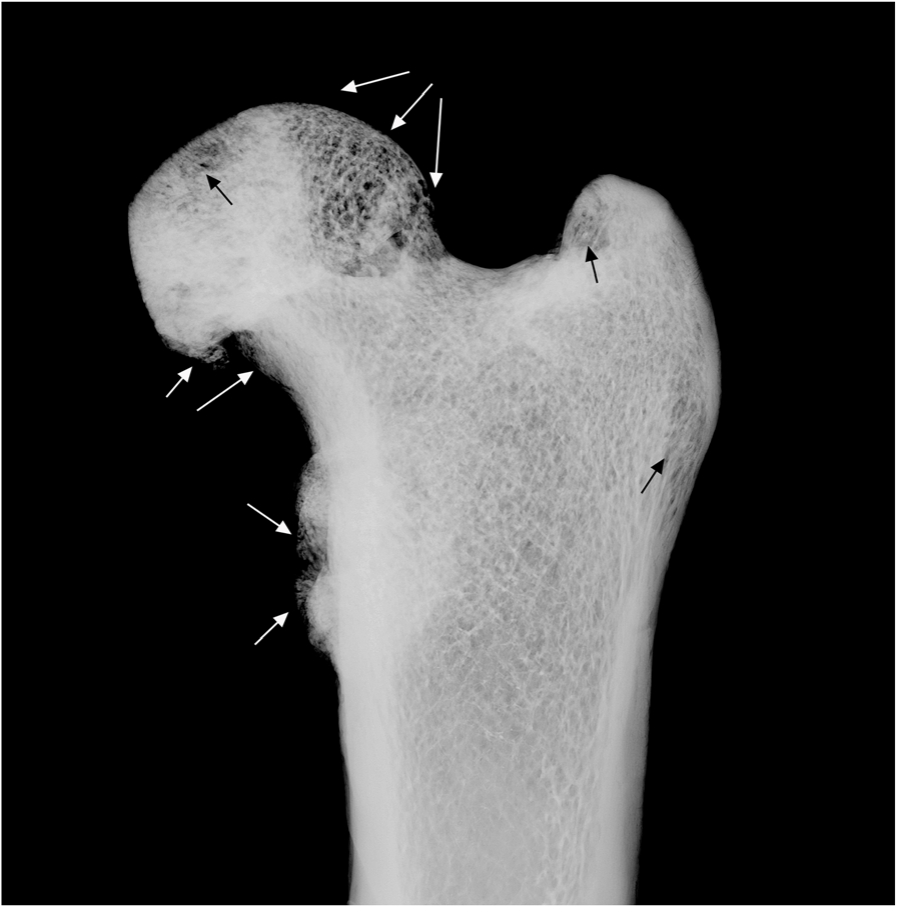
Radiograph of the right femur of *Tamandua tetradactyla* (NPM 069). White and black arrows, radiolucent regions indicating bone remodeling, associated with bone neoformation and bone rarefaction

The proximal epiphysis of the right femur of NPM 069 also has marked signs of osteopathological processes (Fig. 10). The head of the femur is slightly worn and has a yellowish color in the proximomedial region. The distal margin of the femoral head is heavily worn and has an irregularly crenulated shape. Bone resorption is seen inside the *fovea capitis femoris*, with irregular grooves in its inner surface and erosions in its borders, resulting in the expansion of the perimeter of this structure when compared with healthy specimens examined. The femoral neck of the right femur has heavy bone loss and is thinner than in the left femur. The neck of the right femur has an irregular sclerotic lesion medially extending to the proximal portion of the diaphysis, below the lesser trochanter. The proximomedial and posterior edges of the lesser trochanter have protruding and thick bony neoformations. In addition, the surface of the lesser trochanter is irregular and has some small concavities associated with heavy bone thickening. Radiological analysis revealed heavy bone loss in the lateroproximal region of the femoral head, with slightly disordered bone trabeculae and well-marked radiolucent spaces indicating evident bone rarefaction (Fig. 11). Less intense bone loss is also seen in the medial portion of the greater trochanter. The sites of bone erosion in the femoral neck and bone neoformation in the lesser trochanter are evident on radiographic images.

The condition of the right femur of NPM 069 clearly indicates an interaction of the inflammatory processes in this bone with those in the right acetabulum from the same specimen. The lesions in the acetabulum and right femur were tentatively identified as an advanced case of osteoarthritis that possibly affected NPM 069 as age increased.

**Fig. 12:**
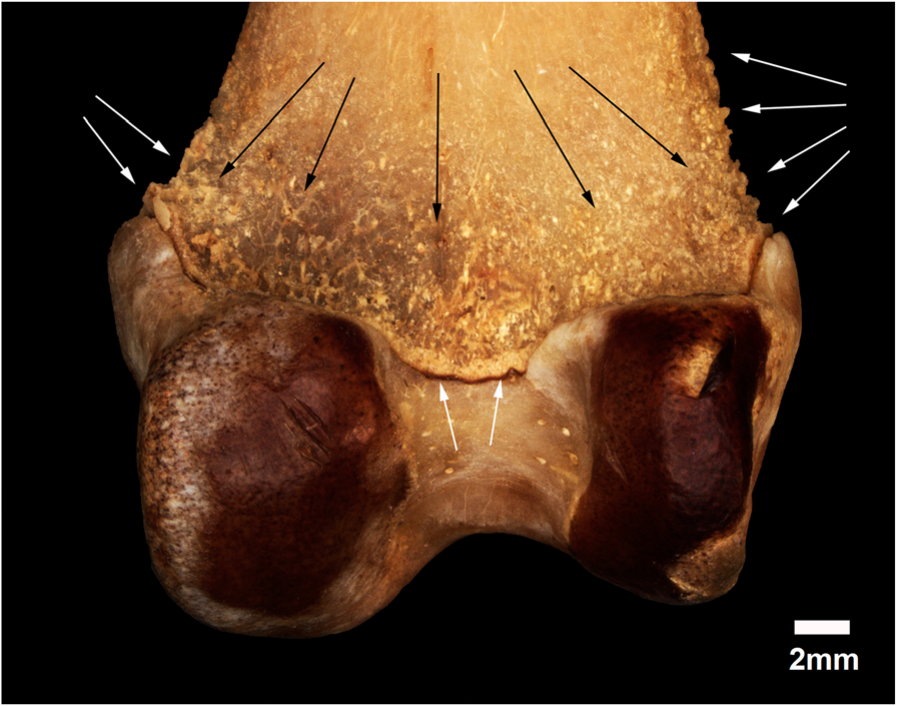
Distal epiphysis of the left femur of *Tamandua tetradactyla* (MN 79028). White arrows, bone remodeling with some osteophytes; Black arrows, Bone erosions

The distal epiphysis of the left femur in specimen MN 79028 has some bone remodeling in the posterior portion similar to that of the proximal third of the humeri of the same specimen (Fig. 12). Some bone remodeling associated with osteophytes is also found in the medial and lateral portions of the distal epiphysis of the left femur.

**Fig. 13:**
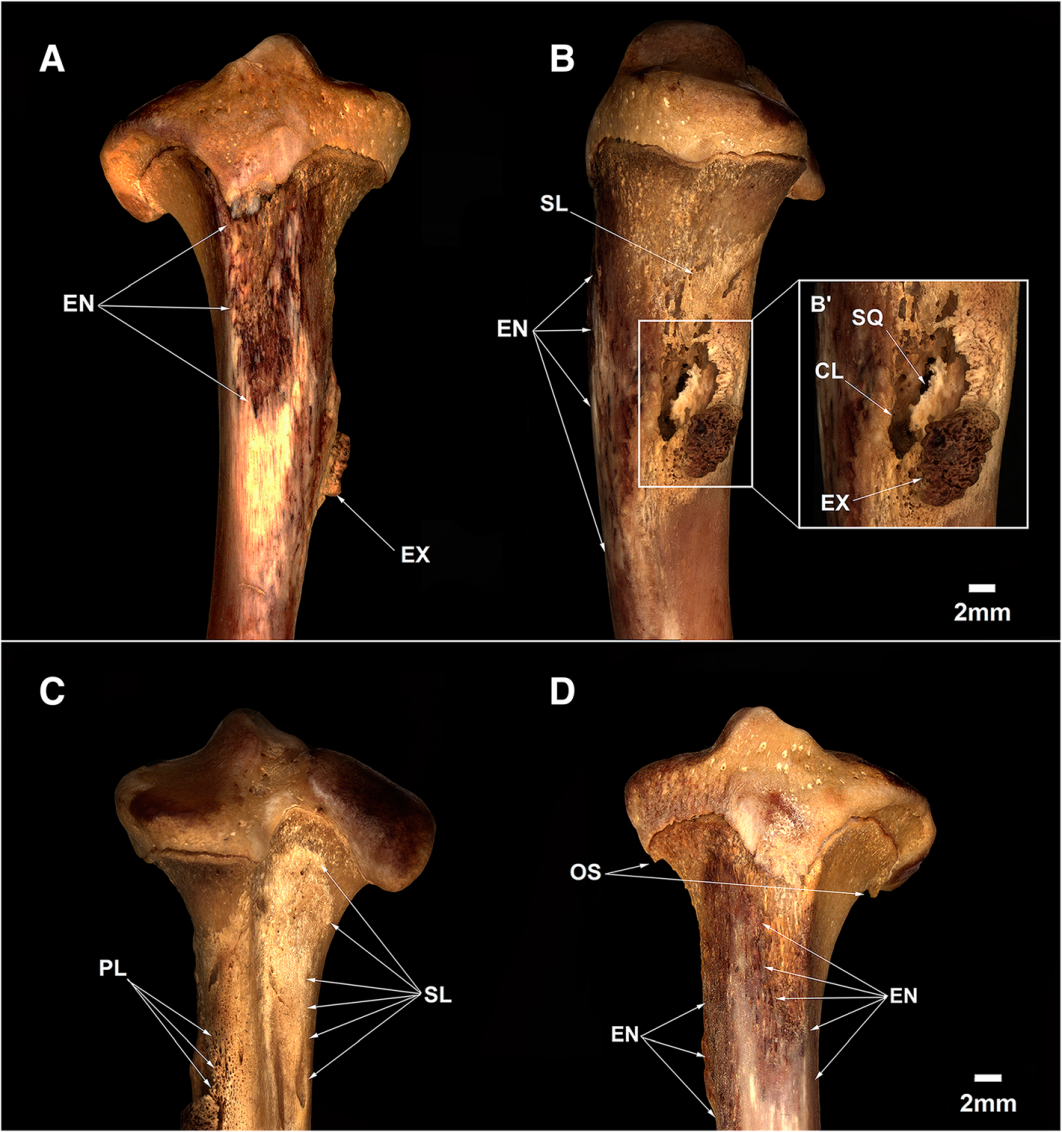
Osteomyelitis and enthesopathy in the tibiae of *Tamandua tetradactyla* (MN 79028). (**a**, **b**, **c**) Proximal third of the right tibia in anterior, medial and posterior views, respectively; **b**’ Osteomyelitis highlighted; **d** Proximal third of the left tibia in anterior view. CL, Cloaca of pus; EN, Enthesopathy; EX, Exostosis; OS, Osteophytes; PL, Porous lesions; SL, Sclerotic lesions; SQ, Sequestrum

The tibiae of MN 79028 have the most severe lesions found. The proximal third of the right tibia has advanced bone destruction in the anterior portion and a marked black color around this region (Fig. 13a).

The proximal third of the right tibia in MN 79028 also shows heavy periosteal and cortical destruction medially (Fig. 13b), resulting in a slightly porotic region, whereas accentuated well-defined sclerotic lesions are present posteriorly (Fig. 13c). Another sclerotic lesion is found anteromedially in the proximal third of the right tibia, more compact than the one seen posteriorly and described above. The bone neoformation that caused the sclerotic lesions in the tibia is apparently indicative of the emergence of an involucrum. The medial portion of the proximal third of the right tibia also has a deep cavity reaching the bone marrow (Fig. 13b), with irregular borders and arranged eccentrically. Inside this cavity there is an isolated fragment of dead bone (sequestrum), as well as evident fistulae and advanced bone destruction possibly resulting from local osteonecrosis (Fig. 13b’).

The left tibia of MN 79028 has similar osteopathological processes, with a heavy darkening of the proximal third in anterior and lateral view, some bone destruction on the surface, and a whitish sclerotic lesion (Fig. 13d).

**Fig. 14:**
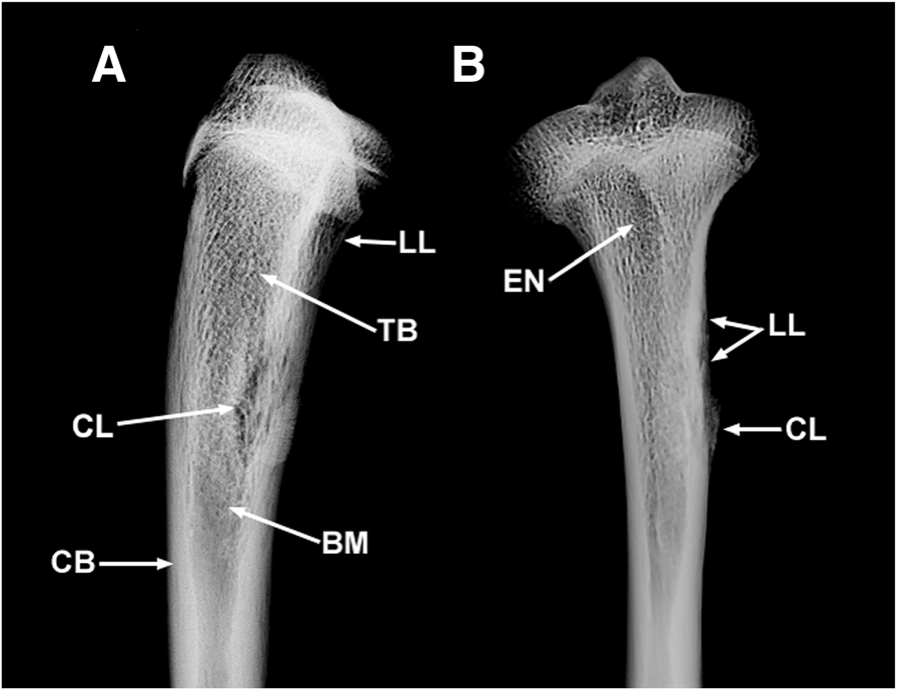
Radiographs of the injured right tibia of *Tamandua tetradactyla* (MN 79028). **a** Right tibia in medial view; **b** Right tíbia in anterior view. BM, Bone marrow; CB, Cortical bone; CL, Cloacae of pus; EN, Enthesopathy; LL, Lytic lesion; TB, Trabecular bone

An involucrum is a layer of new bone growth around the infected bone resulting from the stripping-off of the periosteum by the accumulation of pus within the bone [22], whereas a cloaca is an opening in an involucrum through which pus drains out of the dead bone [20]. The presence of a deep cloaca reaching the bone marrow, sequestrum, and involucrum in the right tibia of MN 79028 is indicative of pyogenic osteomyelitis with advanced destruction of cortical and subcortical tissue, a result of frequent attempts by the body to recover bone mass [20, 23, 24]. The clear radiolucent spaces visualized on radiographic images indicate severe loss of bone tissue in the internal region of the cloaca and in peripheral regions, supporting the identification of the disease in the right tibia of MN 79028 as chronic osteomyelitis (Fig. 14a).

The anterior and lateral portions of the proximal third of the left tibia show no cortical or subcortical bone destruction, and the inflammatory lesion is restricted to the surface of the periosteum (Fig. 14b). The regions examined were possibly affected by advanced enthesopathy that progressed to periostitis as a secondary reaction to this condition.

Anomalous bone growth is seen near the proximal border of the cloaca in the proximal third of the right tibia of MN 79028 (Fig. 13b’). This structure is slightly elliptical-shaped with an irregular external surface corresponding to the morphology of an exostosis [20]. The presence of exostoses in the long bones of other wild mammals (e.g., huemul, kangaroos) has been reported in conjunction with cases of bone trauma, inflammation and, in some samples, intoxication by chemical compounds [25, 26]. The tibial exostosis in MN 79028 is markedly more developed than the exostoses in the scapula from the same specimen. The adjacent regions of this large exostosis showed no osteophytes as occurs in cases of osteoarthritis [22]. The porous and rarefied aspect of the bone surface near the exostosis suggests that the inflammation arising from osteomyelitis spread to the regions adjacent to the cloaca. Thus, it is possible that the drainage of pus to the surface through the cloaca has caused inflammation of the entheses, contributing to the development of this exostosis. Nevertheless, the development of tibial exostosis due to a biomechanical stress event in MN 79028 prior to the development of osteomyelitis was not ruled out, but no feature was found that supported this hypothesis.

## Discussion

**Fig. 15:**
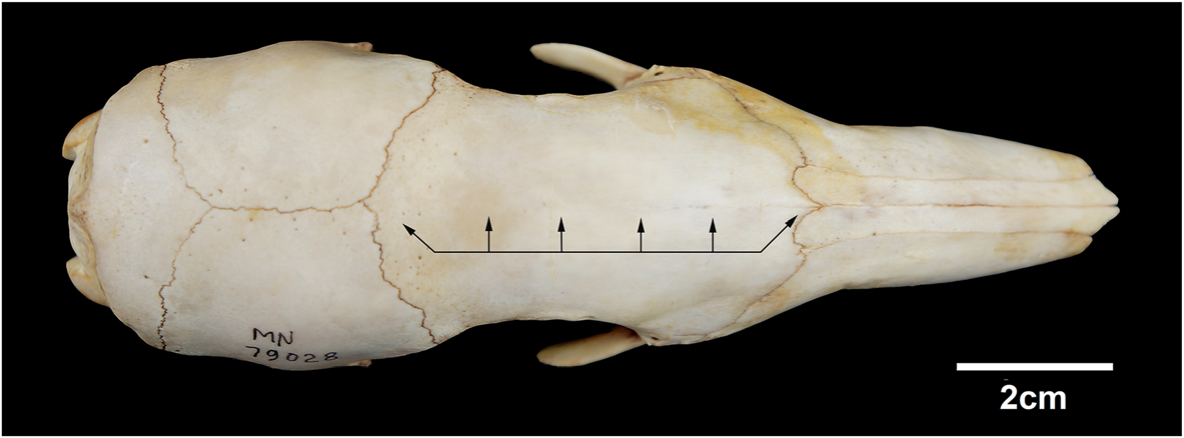
Skull of *Tamandua tetradactyla* (MN 79028). Black arrows, Ossified interfrontal suture of the skull

The condition of the *Tamandua tetradactyla* specimens examined (MN 79028, MN 79287, MZUSP 32329, and NPM 069) enabled the identification of diseases previously unknown for this species, with implications for its biomechanical abilities. The bones of the diseased specimens share some features with all the old *T. tetradactyla* analyzed in this study, such as: fully fused long bone metaphyses; wear on long bone articular capsules; well-developed long bone epiphyses with evident bone thickening; slightly arched long bones diaphyses; and fully ossified interfrontal skull suture (Fig. 15). Considering the probable ontogenetic character of these features, we can infer that the origins of many diseases discussed below are related to the advanced age of the diseased specimens.

The muscles of *T. tetradactyla* are well-developed and likely subject to intense biomechanical forces during locomotion and foraging, mainly in the forelimbs [27]. The lesions identified in the acromion process of the left scapula of MN 79028 (Fig. 1) were likely caused by musculoskeletal interactions. The *acromiotrapezius* muscle is inserted in the acromion process and is responsible for the elevation and dorsoposterior rotation of the scapula. The action of the *acromiotrapezius* in combination with the *serratus magnus* muscle results in intense biomechanical stress in the scapular region of *Tamandua*. The *M. serratus magnus* is responsible for the posteroventral rotation of the scapula, and its conformation in *Tamandua* is distinct from that in other scansorial and arboreal mammals (e.g., *Didelphis*; [27]). In *Tamandua*, the posterior slips of *serratus magnus* are strongly inserted in the dorsal and posterior borders of the scapular angle, whereas in other mammals, mostly only the anterior slips are inserted in the dorsal border of the scapula. The muscle modification in the *serratus magnus* of *Tamandua* enables the protraction and elevation of the glenoid region, aiding in climbing and achieving an upright defensive posture [27]. According to these observations, the acromion of the left scapula of MN 79028 was probably subjected to constant biomechanical stress, especially during elevation of the forelimbs.

The thickening of the distal end of the coracoid was possibly also conditioned by similar biomechanical stress events. The *subscapularis* muscle is arranged in nine multipennate sections and is modified for increased power output in the forelimb. One of the nine sections arise from the anterior border of the scapula and is inserted at the end of the coracoid process and in part of the coracoacromial ligament [27]. Thus, in *T. tetradactyla*, the coracoid process is subjected to great protraction/retraction forces during locomotion [27].

In MN 79028, the biomechanical injuries stemming from use over life probably caused small inflammations in the acromion and coracoid processes of the left scapula, leading to periosteal reactions and the onset of periostitis [22]. The thickening present in some regions of the coracoid and acromion were probably an attempt by the body to recover bone mass, resulting in bone neoformation in some of these regions. The presence of osteophytes in the scapular neck of the left scapula of MN 79028 contributed to the diagnosis of inflammation in this bone.

The deltoid tuberosities of the humeri and the radial ridges of the radii of MN 79028 had inflammatory lesions that provided a better understanding of the osteopathological conditions found in the scapula from the same specimen. The mid-distal regions of the deltoid tuberosities of the humeri of MN 79028 exhibited the greatest periosteal reaction, with bone neoformation, bone remodeling and small, well-defined fistulae indicative of osteopathy. The mediodistal portion of the deltoid tuberosity of the humerus in *Tamandua* corresponds to the insertion area of the *brachioradialis* muscle [27]. This muscle is responsible for elbow flexion in conjunction with the *biceps*, *brachialis*, and *extensor carpi radialis* muscles. The distal portion of the radial ridge, where the *pronator teres* muscle is strongly inserted, was the region of the radii that presented more severe biomechanical lesions in our analysis. [27]. In fact, the *pronator teres* is more developed in *Tamandua* than in other scansorial mammals (e.g., opossum) providing for powerful pronation in the radius [27].

The observations made above suggest that the affected bone areas in MN 79028 were subject to constant biomechanical forces during forelimb elevation, protraction, and pronation. The species included in *Tamandua* are described as animals with frequent scansorial habits [8, 28, 29]. Thus, considering their locomotor habits, lesions stemming from abduction/adduction were expected to be more prevalent in the hindlimbs. However, the bone lesions in MN 79028 are indicative of biomechanical stress during terrestrial locomotion, where specific movements (elevation/protraction/pronation) are often accomplished with the forelimbs. The severe osteomyelitis in the right tibia from specimen MN 79028, combined with its old age, probably contributed to the emergence of these biomechanical lesions.

**Fig. 16:**
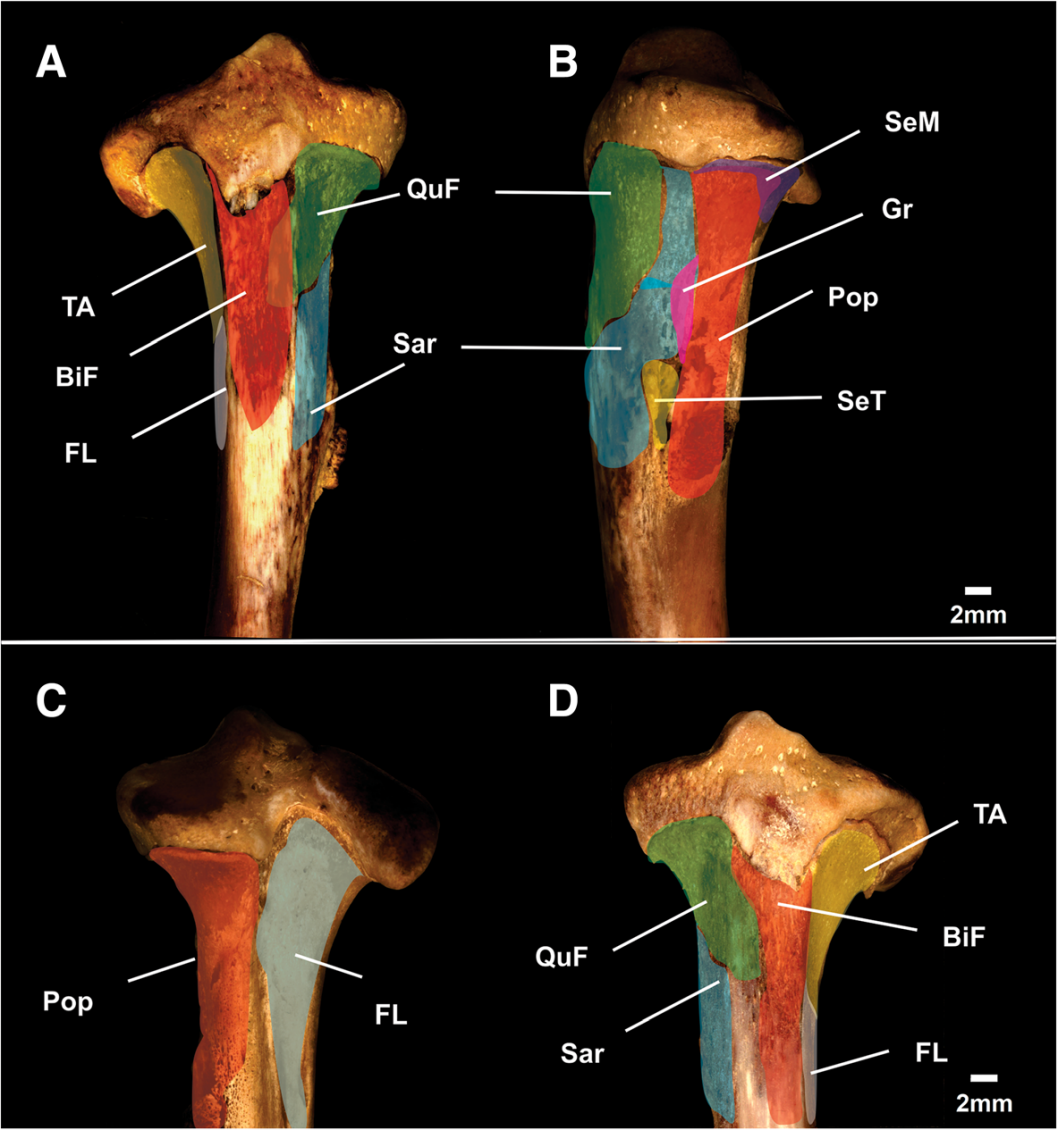
Reconstructions of muscular areas in the tibiae of *Tamandua tetradactyla* (MN 79028). **a**, **b**, **c** Proximal third of the right tibiae in anterior, medial and posterior views, respectively; **d** Proximal third of the left tibiae in anterior view. BiF, Insertion of the *biceps femoris* Muscle; FL, Origin of the *flexor lonqus digitorum* muscle; Gr, Insertion of the *gracilis* muscle; Pop, Insertion of *popliteus* muscle; QuF, Insertion of *quadriceps femoris* muscle; Sar, Insertion of the *sartorius* muscle; SeM, Insertion of the *semimembranosus* muscle; SeT, Insertion of the *semitendinosus* muscle; TA, Origin of the *tibialis anterior* muscle

The tibiae are the largest and strongest of the two lower leg bones, with many powerful muscles that move the foot and lower leg anchored to them. The advanced osteomyelitis identified in the right tibia from specimen MN 79028 probably caused musculoskeletal injuries that impaired its locomotive capacity. Thus, it was necessary to reconstruct the musculoskeletal anatomy of the tibia to determine which muscle groups had likely been affected by infection and to discuss this issue properly. The anatomical reconstruction was based on the muscle origin/insertion marks preserved in the tibiae. The injured regions in the right tibia from specimen MN 79028 correspond to the insertion of the *biceps femoris* (anteriorly), *quadriceps femoris* (proximomedially), *gracilis* (proximomedially), *sartorius* (distomedially), *semimembranosus* (proximomedially), *semitendinosus* (distomedially), and *popliteus* (medially, posteriorly) muscles; and the origin of the *flexor longus digitorum* (distolaterally, posteriorly) and *tibialis anterior* (proximolaterally, anteriorly) muscles (Fig. 16). In mammals, these muscles are responsible for extension (*B. femoris*; *gracilis*; *semitendinosus*; *semimembranosus*) and flexion (*Quadriceps femoris*; *sartorius*) of the hip, extension (*B. femoris*; *Q. femoris*) and flexion (*Gracilis*; *semitendinosus*; *semimembranosus* [as well as the thigh]; *popliteus*) of the knee, and extension (*B. femoris*; *gracilis*; *semitendinosus*) and flexion (*T. anterior*) of the foot [30–33]. Considering that these muscles were constantly interacting with the osteomyelitis sites, it is safe to assume they were affected by severe injuries in this region of the leg, especially with the progression of disease and extrusion of pus to the surface. In addition, some sensory nerves (e.g., *cutaneous ramus* of the femoral nerve, saphenous nerve) were inserted at the lesion site of the right tibia from specimen MN 79028, and it is possible that these nerves were also affected by osteopathological processes (sensu [34]). The prehensile function of the foot was also likely compromised by these lesions, resulting in reduced claw protraction. Such advanced osteopathological lesion was probably chronic and impaired locomotion. In addition, the enthesopathic lesions identified in the humerus, radius, and tibia from specimen MN 79028 were possibly exacerbated by the muscular effort exerted during locomotion. In mammals, enthesopathies should be analyzed with caution, because they may have a biomechanical, age, or weight-bearing origin, or be related to degenerative, inflammatory, or metabolic processes [35]. However, the neoformation, bone loss, and presence of fistulae/cloaca associated with the reported enthesopathies in MN 79028 are evidence of the inflammatory origin of these diseases.

Adult *Tamandua* can reach a total length of 1300 mm and a body mass of up to 7.0 kg (females; [36]). When active, *T. tetradactyla* is scansorial and uses branches for moving and foraging, and often sleeps in trees [37]. Thus, *T. tetradactyla* limbs play an important role in weight-bearing activities. Analysis of the limb bones from specimen MN 79028 indicated that it had a locomotor deficit due to osteopathological processes: its climbing capacity was impaired, and it was forced to use terrestrial locomotion as an alternative because of severe diseases.

The synsacrum in MN 79028, MN 79287, and MZUSP 32329 had thicker regions that are also likely related to biomechanical stress injuries. However, the absence of osteopathological processes in the hindlimbs from specimens MN 79287 and MZUSP 32329 does not support the hypothesis that these lesions were associated with inflammatory diseases. The bone thickening in the synsacrum of these specimens was probably due to lesions caused by cumulative locomotor efforts throughout life whereby the continued action of flexor and adductor muscles (e.g., *sartorius*) contributed to bone neoformation and remodeling (sensu [32, 33, 38]). The presence of bone remodeling in muscle and ligament insertion sites in the femora and humeri from specimen MN 79028 are suggestive of overuse injuries, with enthesopathies less severe than those observed in other bones from the same specimen.

The traces of inflammatory processes in the right femoroacetabular region from specimen NPM 069 provides strong evidence of an advanced osteoarthritis case. The intense erosion in the femoral neck of the right femur and extensive bone remodeling in the internal and external aspects of the acetabulum of the right synsacrum are indicative of an advanced osteopathological process that probably became more aggressive with advancing age.

Based uniquely on the analysis of the external and internal morphology of the bones of MN 79028, we were unable to identify the causative pathogen of osteomyelitis. However, molecular and microbiological investigations may provide an indication of the pathogen involved.

Bone diseases have been examined in xenarthran fossils in recent years (e.g., [39, 40]), but knowledge about extant species is scarce and therefore impairs valid anatomical comparisons. Moreover, few studies have examined museum specimens. Thus, bone diseases and their causes in xenarthrans remain largely unknown. In other vertebrate groups, biological collections are often used to track infectious diseases and potential reservoir species [41]. For instance, analysis of museum rodent specimens helped establish the relationship of these specimens and their large populations with the increased risk of human infection with *Hantavirus* (Bunyaviridae) in Southwestern United States [41–44]. Similarly, analysis of influenza virus preserved in bird specimens from the Smithsonian Institution, which were compared with tissue samples from humans infected in the 1918 global influenza outbreak, revealed a greater similarity of the virus that caused this pandemic with the swine and human viruses than with the avian influenza virus [41, 45, 46]. Thus, the analysis of bone diseases in anteaters not only provides informative material about Xenarthra that can be useful for animal health studies, including paleopathological researches, but also allows for broader investigations that may reveal pathogens associated with the group and other mammals, including humans.

## Conclusion

Bone diseases were diagnosed in four *Tamandua tetradactyla* specimens, the most severe of which were identified in specimens NPM 069 and MN 79028. Specimen NPM 069 had advanced femoroacetabular osteoarthritis, whereas MN 79028 had multiples lesions, the most aggressive of which was chronic pyogenic osteomyelitis in the right tibia, the first case described for *Tamandua*. Other diseases were found in MN 79028, including biomechanical stress lesions in the left scapula and femora, in addition to enthesopathies in the humeri, radii, and tibiae. The affected sites in the appendicular skeleton of this specimen indicate that the animal probably had difficulty climbing and was restricted to terrestrial locomotion, resulting in musculoskeletal overuse injuries caused by repeated forelimb movements during locomotion (forelimb elevation/protraction/pronation), which were further complicated by its advanced age. Bone neoformation caused by biomechanical stress was also identified in the synsacrum from specimens MN 79028, MN 79287, and MZUSP 32329.

The description of bone diseases in Vermilingua provides not only data unique to the group, enabling a more accurate diagnosis of bone diseases in anteaters, but also useful information for pathology of extant and fossil xenarthrans. In addition, this study has implications for animal welfare, particularly for wild and captive vermilinguans. Finally, our findings highlight the importance of biological collections as sources for animal health studies [44, 47].

## Methods

### Material examined

The bone samples of *Tamandua tetradactyla* surveyed for pathological lesions include 64 museum specimens with almost complete postcranial skeletons. This material includes skeletons from males and females of different ages and from several localities in Brazil, which are deposited in the following Brazilian institutions (Table 1): Mammal collection at the Institute of Biodiversity and Sustainability at the Federal University of Rio de Janeiro (NUPEM/UFRJ), Macaé, Rio de Janeiro (NPM); Mammal collection at the National Museum, Federal University of Rio de Janeiro, Rio de Janeiro (MN); collection of extant mammals at the Museum of Earth Sciences of the Company of Mineral Resources Research, Rio de Janeiro (MR); Mammal collection at the Museum of Zoology at the University of São Paulo, São Paulo (MZUSP), Mammal collection at the Federal University of Minas Gerais (UFMG), Mammal collection at the Museum (MCN; LOM) of Pontifical Catholic University of Minas Gerais (PUC-MINAS).

**Table 1:** Specimens of *Tamandua tetradactyla* examined according to sampling locality

Museum voucher	Sex	Age class	Collection location
MZUSP 7336	Female	Adult	Acre, Brazil
MN 3846	Male	Adult	Anápolis, Goiás, Brazil
MN 4538	Female	Young	Anápolis, Goiás, Brazil
MN 5059	Male	Adult	Anápolis, Goiás, Brazil
MN 5068	Female	Young	Anápolis, Goiás, Brazil
MN 5069	Female	Young	Anápolis, Goiás, Brazil
MZUSP 35104	Undefined	Old	Salinas, Chapada dos Guimarães, Mato Grosso, Brazil
MN 5056	Female	Adult	Maracajú, Mato Grosso do Sul, Brazil
MN 5061	Male	Adult	Maracajú, Mato Grosso do Sul, Brazil
133 MR	Undefined	Old	Antonina, Paraná, Brazil
MN 63464	Female	Adult	São Raimundo Nonato, Piauí, Brazil
MN 63508	Female	Adult	São Raimundo Nonato, Piauí, Brazil
MN 63520	Undefined	Adult	PI 140, American Man Museum Foundation, Piauí, Brazil
UFMG 4575	Undefined	Subadult	Buriti dos Montes, Piauí, Brazil
UFMG 3984	Undefined	Infant	Complexo Ponta da Madeira, São Luís, Maranhão, Brazil
UFMG 3986	Undefined	Infant	Complexo Ponta da Madeira, São Luís, Maranhão, Brazil
MZUSP 21329	Male	Old	Cachoeira do Espelho, Xingu River, Pará, Brazil
MZUSP 20002	Female	Young	Fordilândia, Pará, Brazil
MN 67817	Undefined	Young	Chapada Diamantina, Catolés, Abaíra, Bahia, Brazil
MN 73484	Female	Subadult	Hydroelectric plant of Irapé, Berilo, Minas Gerais, Brazil
MN 79027	Male	Adult	Red house, area of exploitation of the hydroelectric plant of Anta/Simplício (AHE), Minas Gerais, Brazil
MN 79028	Female	Old	Construction site of the hydroelectric plant of Anta/Simplício (AHE), Minas Gerais, Brazil
MN 79171	Undefined	Young	Juiz de Fora, Minas Gerais, Brazil
MN 79446	Undefined	Young	Matias Barbosa, Minas Gerais, Brazil
MN 79287	Male	Old	Matias Barbosa, Minas Gerais, Brazil
MN 79179	Undefined	Adult	Simão Pereira, Minas Gerais, Brazil
MN 79135	Female	Young	Simão Pereira, Minas Gerais, Brazil
MCN-M 2787	Undefined	Infant	Nova Lima, Minas Gerais, Brazil
MCN-M 2617	Undefined	Old	Governador Valadares, Minas Gerais, Brazil
MCN-M 2101	Female	Young	Rio Piracicaba, Minas Gerais, Brazil
CGCV 01	Undefined	Old	Conceição do Mato Dentro, Minas Gerais, Brazil
LOM 338	Undefined	Subadult	Conceição do Mato Dentro, Minas Gerais, Brazil
LOM 378	Female	Subadult	Conceição do Mato Dentro, Minas Gerais, Brazil
UFMG 4526	Undefined	Young	Mariana, Minas Gerais, Brazil
UFMG 4338	Female	Young	Santa Luzia, Minas Gerais, Brazil
UFMG 6077	Undefined	Young	Nova Lima, Minas Gerais, Brazil
UFMG 6165	Female	Young	Belo Horizonte, Minas Gerais, Brazil
MN 5515	Male	Adult	São João de Petrópolis, Espírito Santo, Brazil
MN 5507	Female	Young	Córrego de Santa Teresa, Espírito Santo, Brazil
MN 79270	Undefined	Adult	Xerém, Duque de Caxias, Rio de Janeiro, Brazil
MN 26631	Undefined	Adult	National Biological Reserve of Poço das Antas, Silva Jardim, Rio de Janeiro, Brazil
MN 79513	Female	Young	Br 040 Km 20, Três Rios, Rio de Janeiro, Brazil
MN 79361	Male	Young	Petrópolis, Rio de Janeiro, Brazil
MN 832354	Undefined	Young	Br 040, Km 91, Rio de Janeiro, Brazil
MN 79118	Undefined	Subadult	Br 040 Km 75, Araras, Petrópolis, Rio de Janeiro, Brazil
NPM 002	Female	Adult	Road Amaral Peixoto, location near to Municipal Park of Rio das Ostras, Rio de Janeiro, Brazil
NPM 1317	Undefined	Adult	Collected by the Department of Environment and Sustainability of Macaé Prefecture (SEMA), Rio de Janeiro, Brazil
NPM 289	Female	Adult	Municipal Natural Park Fazenda Atalaia, Macaé, Rio de Janeiro, Brazil
NPM 472	Female	Subadult	Br 101, Biological Reserve União, Casimiro de Abreu, Rio das Ostras, Rio de Janeiro, Brazil
NPM 069	Female	Old	Biological Reserve União, Casimiro de Abreu, Rio das Ostras, Rio de Janeiro, Brazil
NPM 488	Undefined	Adult	BR 101, Furnas, Rio de Janeiro, Brazil
NPM 296	Female	Subadult	Br 101, Biological Reserve União, Casimiro de Abreu, Rio das Ostras, Rio de Janeiro, Brazil
NPM 539	Female	Old	Br 101, Km 187, location near to Biological Reserve União, Casimiro de Abreu, Rio das Ostras, Rio de Janeiro, Brazil
MZUSP 35104	Undefined	Old	Rio de Janeiro, Brazil
MZUSP 22357	Undefined	Old	Rio grande do Sul, Brazil
MZUSP 32620	Male	Subadult	Municipal Zoological Park Quinzinho de Barros, Sorocaba, São Paulo, Brazil
MZUSP 31990	Undefined	Old	Ecological Station of Jataí, Luís Antônio, São Paulo, Brazil
MZUSP 32329	Undefined	Old	Zoological Park of São Paulo, Brazil
MCN-M 76	Male	Old	Zoological Park of Belo Horizonte, Brazil
MCN-M 2379	Undefined	Old	Zoological Park of Belo Horizonte, Brazil
MCN-MZ 12	Undefined	Old	Zoological Park of Belo Horizonte, Brazil
MCN-MZ 219	Undefined	Subadult	Zoological Park of Belo Horizonte, Brazil
MN 43804	Undefined	Young	Undefined
LOM 481	Undefined	Young	Undefined

The specimens with bone diseases were collected from different localities: MN 79028 was found dead on January 22nd, 2010 in a small fragment of Atlantic Forest under anthropogenic pressure from the construction of the Anta/Simplício hydropower plant in the Paraíba do Sul River, state of Minas Gerais, Brazil; MN 79287 was collected in Matias Barbosa, Minas Gerais, Brazil; NPM 069 was collected in the União Biological Reserve, Casimiro de Abreu, Rio das Ostras, state of Rio de Janeiro, Brazil; MZUSP 32329 was donated to MZUSP by the Zoological Park of São Paulo, São Paulo, Brazil.

### Anatomical and pathological terms

The diagnoses and descriptions of the bone diseases observed in *T. tetradactyla* followed procedures used in the current literature [20–22, 24, 48]. These studies described and analyzed skeletal diseases in other vertebrate groups, including humans, and were used as reference due to a paucity of information on bone injuries of anteaters.

The anatomical terminology is based on osteological studies of the postcranium of anteaters [3, 27] and other vertebrates [31–33, 49].

The abbreviations for the muscles after the first citation follow the accepted terminology used in animal anatomy studies (muscle = M.; muscles = Mm. [50]).

### Macrophotographs, microphotographs and radiographs

Macrostructure photographs were taken using a Sony Nex F3 16.1 MP digital camera, whereas microstructures were observed using a Leica M205C binocular stereomicroscope at the Laboratory of Herpetology (MN/UFRJ). Radiographs were taken in a Faxitron cabinet at the Radiography Laboratory of the Department of Vertebrates, National Museum, Federal University of Rio de Janeiro (MN/UFRJ) and a Faxitron LX-60 cabinet at the Integrated Zoology Laboratory of the Institute of Biodiversity and Sustainability at the Federal University of Rio de Janeiro (NUPEM/UFRJ). Photographic, micrographic, and radiographic images were prepared for publication in Adobe Photoshop CC 2015 and Picasa software.

## Abbreviations

M: Muscle
MCN/LOM: Mammal collection at the Museum of Pontifical Catholic University of Minas Gerais, Brazil
Mm: Muscles
MN: Mammal collection at the National Museum, Federal University of Rio de Janeiro, Rio de Janeiro, Brazil
MR: Collection of extant mammals at the Museum of Earth Sciences of the Company of Mineral Resources Research, Rio de Janeiro, Brazil
MZUSP: Mammal collection at the Museum of Zoology at the University of São Paulo, São Paulo, Brazil
NPM: Mammal collection at the Institute of Biodiversity and Sustainability at the Federal University of Rio de Janeiro (NUPEM/UFRJ), Macaé, Rio de Janeiro, Brazil
UFMG: Mammal collection at the Federal University of Minas Gerais, Brazil
